# Realtime physical simulator for virtual reality sailing by patients with spinal cord injury: an innovative voyage

**DOI:** 10.12688/healthopenres.13582.1

**Published:** 2024-02-16

**Authors:** Albert C Recio, Steven A Stiens, Marjorie Morgan, Shalini Selvarajah, Amna C Mazeh, Mark D Habgood, Norman R Saunders

**Affiliations:** 1 Department of Physical Medicine & Rehabilitation, Johns Hopkins University School of Medicine, Baltimore, MD, USA; 2 The International Center for Spinal Cord Injury, Hugo W. Moser Research Institute, Kennedy Krieger Institute, Baltimore, MD, USA; 3 Downtown Sailing Center, Baltimore Museum of Industry, Baltimore, MD, USA; 4 Department of Physical Medicine and Rehabilitation, Geisinger College of Medicine, Scranton, PA, USA; 5 Pharmacy Quality Alliance, Wake Forest University, Creedmore, NC, USA; 6 Department of Biochemistry and Pharmacology, University of Melbourne, Parkville, Victoria, 3010, Australia; 7 Department of Neuroscience, Central Clinical School, Monash University, Melbourne, Victoria, 3004, Australia

**Keywords:** Sailing simulator, sailing techniques, spinal cord injury, rehabilitation, on-water sailing, quality of life, self-esteem, psychological health.

## Abstract

**Background:**

The aim of this study was to explore whether sail training using a VSail® simulator would allow people with spinal cord injuries (SCI) to learn to sail in a safe controlled environment and then sail competently on the water in wind of moderate strength (12 knots). A battery of physical tests and questionnaires was used to evaluate possible improvements in health and well-being as a consequence of participation in the trial.

**Methods:**

Twenty participants were recruited with the assistance of their physicians from The International Center for Spinal Cord Injury, Kennedy Krieger Institute. Inclusion criteria were SCI >6 months previously, medically stable, with no recent (1 month or less) inpatient admission for acute medical or surgical issues. All neurological SCI levels (C1-S1) were eligible. All subjects followed a programme of instruction leading to mastery of basic sailing techniques (steering predetermined courses, sail trimming, tacking, gybing and mark rounding).

**Results:**

Not all participants completed the study for various reasons. Those that did were seven males and six females, six with tetraplegia and seven with paraplegia. The mean age was 45 years (23 to 63) and the average time since injury was 14.7 years (2 to 38 years). At the end of the course subjects were able to perform the sailing maneuvers and navigate a triangular racecourse on the simulator’s display in 12 knots of wind within a pre-set time. At 6 weeks post completion of training most subjects showed a decrease in depression, physical and social limitations, and an improvement in physical tests. These improvements were maintained or increased in most participants by 12 weeks, but not others.

**Conclusions:**

The primary objective of the trial was achieved as all participants who completed the VSail® training were able to sail on the water at the Downtown Sailing Center in Baltimore.

## Plain Language statement

Spinal cord injury can produce a variety of life-limiting chronic impairments, particularly as many occur in young adults. It affects the injured individual, their family, friends and society. Clinical care has improved substantially over the past decades allowing most with spinal injuries to have a normal life span. But many have difficulty in adjusting to the limitations of their new life and are often quite socially isolated. Sailing is usually considered out of reach to most people unless they have a connection through family or friends. It is generally viewed as elitist, expensive and at times dangerous. A view that probably stems from the publicity given to high profile events such as the Americas Cup or long-distance yachting competitions. However, small sailboat sailing is much more available. The problem for even able-bodied people is lack of access to an activity that does appear to carry some risks. For people with spinal injuries sailing seems even more daunting. The aim of this project is to investigate whether use of real time virtual sailing simulators can teach people with spinal cord injury to sail in a safe controlled environment and then easily transition to sail safely and competently on the water. In addition, this project was designed to evaluate the effects on physical and psychological health as well as effects on morale and self-esteem.

The study recruited 20 people from the Kennedy Krieger Spinal Institute. They undertook a standard simulator training protocol involving 12 one-hour sessions. For mainly heath-related reasons not all participants completed these sessions. However, all of the 13 participants who completed the simulator training were able to sail in Hansa dinghies in Baltimore Harbor. Each individual showed improvement in most of the physical tests and in a Quality-of-Life Questionnaire and the Veterans RAND 36-Item Health Survey.

## Abbreviations

AIS, ASIA Impairment Scale; ASIA, American Spinal Injury Association; ISNCSCI, International Standards for Neurological Classification of Spinal Cord Injury; QoL, quality of life; RPE, rate of perceived exertion; SCI, spinal cord injury; C, cervical; T, thoracic; L, lumbar; S, sacral; VR, Veterans Rand.

## Introduction

Person-centred rehabilitation requires a comprehensive assessment of impairments as well as assets, resources, and passions that will drive the process ( Stiens *et al.*, 2013; Stiens *et al.*, 2024). The urgency to promote recovery from spinal cord injury (SCI) in modern rehabilitation requires early comprehensive intervention; this includes modalities that enable critical adaptive skills, reinforce compliance and demonstrate options for life satisfaction. Goals focus on enjoyment and positive expectations for patients. As a result, robotic rehabilitation has been developed to deliver intensity with sufficient repetitions, and stretching resistance to provide refinement of performance with greater efficiency ( Lozano-Berrio *et al.* 2022).

Despite the barriers presented by hospitalized care, the fullest function within the least restrictive environment is a required emphasis ( Stiens & Shamberg, 2024). To achieve this, interdisciplinary teams need to equip patients with the most effective adaptive devices and expose them to environments that minimize their impairments. As a result, adaptive aquatics for example has been incorporated into facility design and treatment programs ( Rojhani *et al.*, 2017).

Adjusting an interface for the patient to work against to achieve strength, endurance and skill produces the desired outcomes. The best environmental interface mimics situations in the real world, promoting carry over to self-sustaining life activities. Effective robotics provide a pertinent connection for exercise that can be linked to a desired activity to be eventually performed in the natural environment. Current trends in therapy include utilization of robotics to active desired activities that are termed activity-based restoration ( Dolbow *et al.*, 2015). Virtual reality systems alone generate psychological benefits for patients in rehabilitation therapy ( Chen *et al.*, 2009; de Araújo *et al.*, 2019; Leemhuis *et al.*, 2021). A potentially important intervention for SCI and other disabilities involves the combination of virtual reality ( de Araújo *et al.*, 2019; Leemhuis *et al.*, 2021) and robotics ( Lozano-Berrio *et al.*, 2022). The VSail® sailing simulator was first designed to provide novice sailors an introduction activity that reduces the complications and unpredictability of learning to sail on the water ( Autry & Anderson, 2020; Binns *et al.*, 2011; Mooney *et al.*, 2009; Recio *et al.*, 2013). Rehabilitation clinicians have used the VSail® Trainer to overcome barriers in the hospital setting and bring patients an opportunity to acquire appropriate skills in anticipation of sailing on open water ( Manzanares *et al.*, 2021; Manzanares *et al.*, 2023; Recio *et al.*, 2013)

Small boat, open water sailing by people with physical, social or psychological disabilities, greatly magnifies self-esteem and enhances general health ( Anderson, 2015; Carta *et al.*, 2014; Jenkin & Wilson, 2009; Salvatoni *et al.*, 2003; Sancassiani *et al.*, 2017). In addition, sailing is a family recreational activity; learning to sail improves family relations and reintegration into the community. For the competitively minded, the worldwide organization Sailability World, Inc. promotes sailing for those with disabilities. With suitably adapted sailboats, people with disabilities compete on equal terms with the able-bodied. Adaptive sailing has the potential to be a potent early interdisciplinary rehabilitation activity if used more widely.

Sailing is an attractive sport that can be pursued at recreational and competitive levels, substantiating valuable interventions and outcomes. Benefits of sport and recreational activities for people with disabilities, are well recognized with measures of health and quality of life. Yet, there remains a lack of options for most people living with disabilities, especially the young. We conducted an introductory sailing program using the VSail® Sailing Simulator.

We recognized that participation in sailing by people with disabilities, particularly in small sailboats, is expected to produce positive outcomes in self-esteem and general health of participants. However, major hurdles for people with no experience with sailing, even those without disabilities, are perceptions that sailing is dangerous and expensive. We expected, real time “ride-on” sailing simulators would have the potential to bridge the gap between dry land and on-water sailing by providing a realistic simulation. The simulator is safe and easily supervised; novice aspiring sailors can easily and systematically learn the required skills before taking boats afloat. The tasks of sailing on a simulator can take place under specified conditions (e.g., wind strength) and be taught specifying particular skills (e.g., steering, sail trimming) in a way that is not possible on the water where there are a large number of simultaneous interacting variables. These individual capabilities can then be mastered individually and sequentially assembled so that the novice sailor rapidly achieves a degree of competence that would take much longer on the water.

Pilot trials in Australia, New Zealand, Spain and the US have successfully used virtual simulation technology ( Manzanares *et al.*, 2021; Manzanares *et al.*, 2023; Recio *et al.*, 2013; Saunders *et al.*, 2016) to introduce people with severe disabilities to sailing on dry land, with a successful transition to on-water competitive and recreational sailing. Remarkably, one patient progressed to compete in a World Championship event in July 2010 and qualified for the 2012 Paralympics, less than three years after her initial simulator training experience! As considered in the Discussion these early trials provided a range of evidence on the effectiveness of virtual sailing technology in providing an access pathway to on-water sailing and improve morale and reintegration into society.

The aim of this project was to establish protocols for effective use of a sailing simulator by people with disabilities and then to conduct a pilot trial to test four hypotheses: (1) That the use of the sailing simulator technology enables people with SCI to learn the skills required to sail on the water in a safe, non-threatening environment. (2) Teaching patients with SCI to sail using simulator technology will provide them with a safe, effective introduction to a healthy, environmentally-friendly, lifelong sport and recreation. (3) Sailing will produce measurable improvements in participants’ physical health and (4) Sailing will improve psychological well-being, including self-esteem, morale, and optimism about the future. 

## Methods

Participants in this pilot trial were a group of 20 subjects aged 18–80 years, with a diagnosis of SCI, recruited from theas outpatients at Kennedy Krieger Institute. Each subject was assessed medically, and with questionnaires to establish baselines for evaluation of quality of life and self-esteem. Before the start of the study, all procedures were approved by Johns Hopkins Institutional Review Board (JHH IRB - NA_00044093). Recruitment was performed in-person through conferring with physicians and staff to determine which patients fitted the eligibility criteria. The eligibility criteria were (a) chronic injury, > 6 months since the SCI (b) ASIA (American Spinal Injury Association) Impairment Scale (AIS) neurological levels (C1-S1) (c) medically stable, with no recent (1 month or less) inpatient admission for acute medical or surgical issues. All participants gave written consent to take part in the study (consent form deposited in Extended data).

### Experimental procedure overview

This within-subject pilot study enrolled 20 chronic (more than 6 months) SCI patients as per recruitment matrix in Table 1. In addition to their previously designed outpatient rehabilitation regimens, the subjects engaged in a virtual reality sailing program (VSail®) one hour per week for 12 weeks. Before starting, the subjects were assessed using a baseline battery of physical and neurological indicators. These tests were repeated after 6 weeks and 12 weeks of training. During each training visit, outcomes were assessed by the physical therapist ( Figure 2).

**Table 1.** Spinal injury classification of participants in the trial. Spinal level of lesion and degree of completeness of lesion indicated.

	High Tetra (C1-C5)	Low Tetra (C6-C8)	High Para (T1-T6)	Low Para (T7-T12)	Cauda Equina (L1-S1)
Motor Complete (A-B)	2	2	2	2	2
Motor Incomplete (C-D)	2	2	2	2	2

### Study intervention

**Figure 1.**
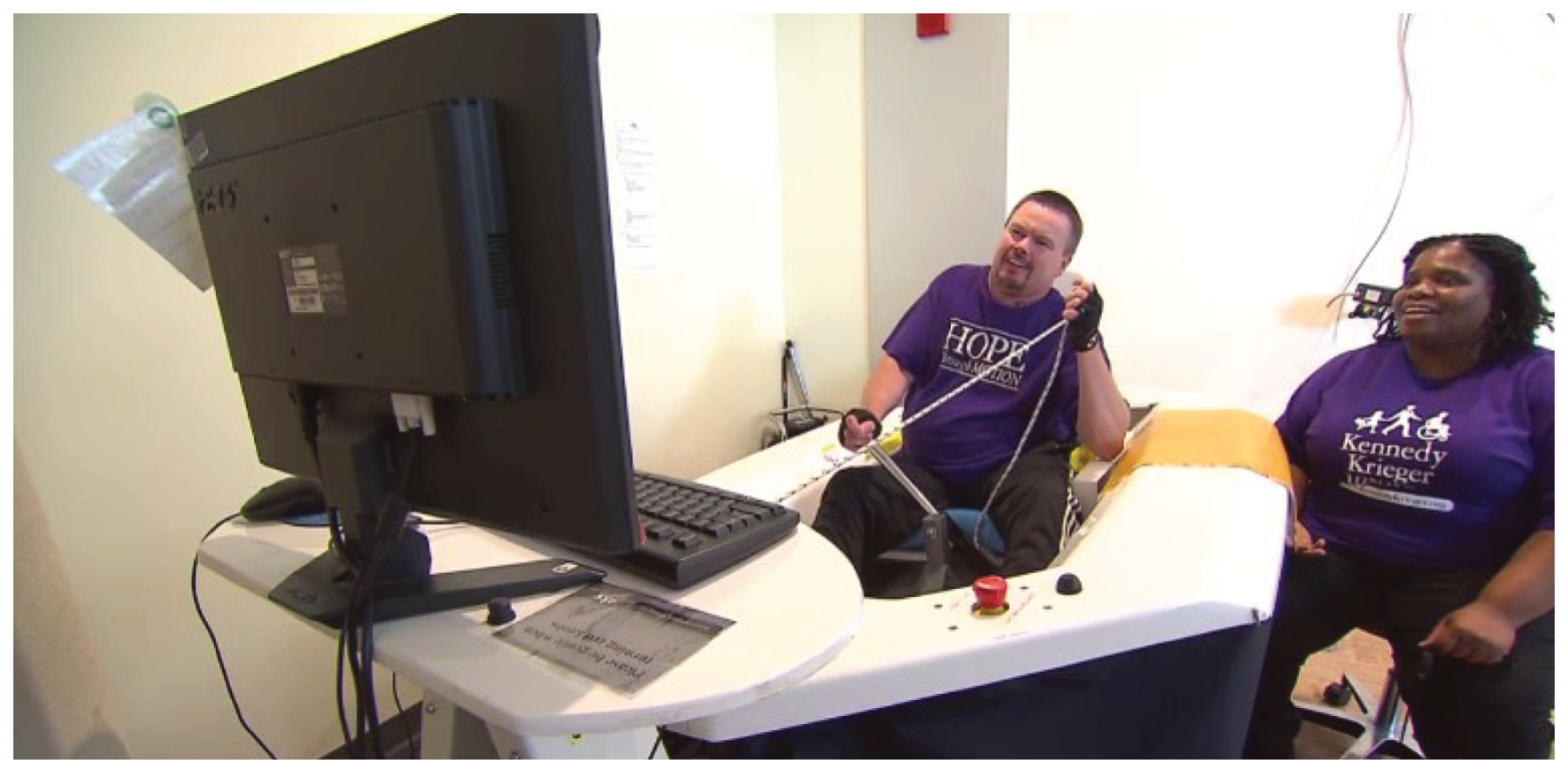
VSail® simulator. Consists of a cockpit and seat for the sailor. The controls, joystick to steer, mainsheet (rope) to adjust sail, are the same as in a Hansa Dinghy. The sailor sees an image of the sailboat on the screen. Sensors on the simulator respond to changes in movement of cockpit, steering and sail control. This information is fed through a computer and displayed on the screen.

All activities were conducted by a physical therapist familiar with the VSail® Trainer. While undergoing the virtual sailing training, the subjects used the VSail® Simulator in Figure 1 ( Virtual Sailing Pty Ltd, Melbourne, Australia). The VSail® Simulator was set in the Access/Hansa Dinghy mode, with a simulation of the Hansa Liberty. The VSail® sailing parameters were adjusted to the subject’s skill set and progression in the training program. Adjustments were made in wind speed from minimum (8 knots) to moderate strength (12 knots), a pneumatic ram (simulating the heeling of the hull) was initially off until steering and controlling the sail were mastered; heeling was then activated.

The subjects transferred to the VSail® cockpit and were secured with waist and shoulder straps in a cushioned rotating chair. Following the trainer’s instruction, the subject sails the simulator around virtual courses displayed on a computer screen, using a joystick to control the rudder angle and a mainsheet rope to control the set of the sail. All subjects followed a course of instruction (see Extended data) leading to mastery of basic sailing techniques such that at the end of the course the subjects will be able to perform a series of standard sailing maneuvers and navigate competently a triangular race course on the simulator’s display in 12 knots of wind within a preset time.

While training, the subjects were assessed for fatigue (17 or over on the Rate of Perceived Exertion (RPE) scale), symptoms and signs of Autonomic Dysreflexia (headache, sweating, facial flushing, goose bumps/piloerection, irritability, crying, sudden and profuse sweating above injury level, intense head/neck tingling systolic blood pressure ≥140 mmHg), and symptoms of orthostatic hypotension (dizziness, light headedness, palpitations, sudden vision loss, profuse sweating systolic blood pressure ≤100 mmHg). 

### Study assessments

**Figure 2.**
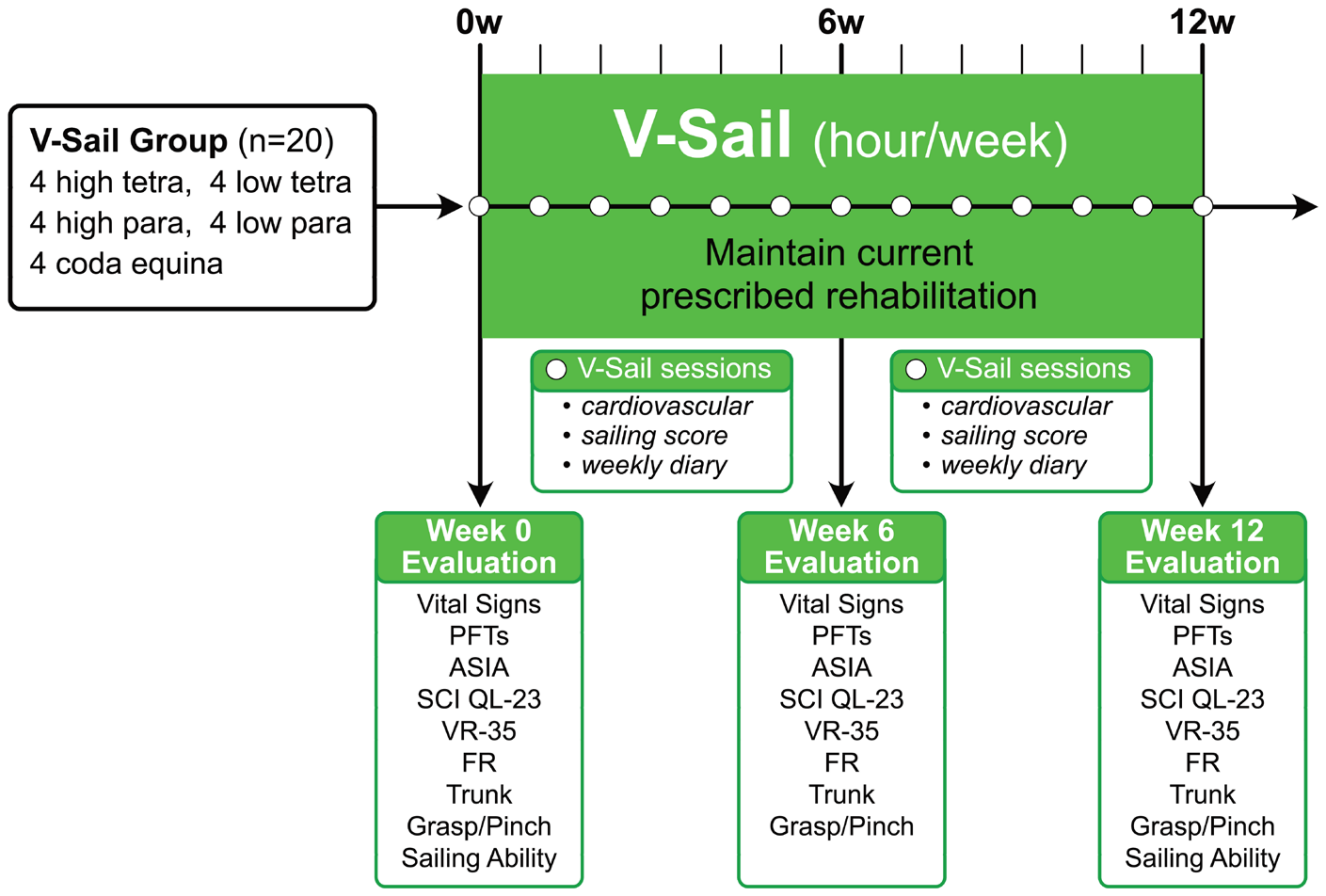
Study timeline. Number of participants and SCI levels shown. Evaluations made at start, 6 weeks and 12 weeks indicated.

This pilot study included a combination of pre- and post-qualitative assessments ( Figure 2). The data collected are shown in the Underlying data.

All subjects were assessed using the **ASIA** (American Spinal Injury Association) Impairment Scale per ISNCSCI test. The ASIA examination assesses motor function of 10 key muscles that represent spinal cord levels and light touch and pinprick sensation at 28 key points within dermatomes on each side of the body. It is used to classify injury level and impairment severity ( Table 1).

Balance was assessed using the **Functional Reach (FR) Test**. This test is used to evaluate trunk balance. This test is performed by patient reaching forward as far as they can while seated in their wheelchair. The distance travelled by the top of the reference shoulder is measured. The forward and lateral reach were also assessed on all subjects. The level of trunk activation was also assessed by asking the patient to perform a sit-up from the supine position. Using landmarks such as sternal angle, nipple line, supra-sternal notch and ribs, the lowest level of trunk activation was determined.

The physical assessments that were completed are as follows:

**Vital signs**: There are no known risks associated with this measurement.

**Pulmonary Function Test** (PFTs): The risk is minimal for most people. Since the test involves some forced breathing and rapid breathing, patients may have some temporary shortness of breath or light-headedness. Patients breathe through a tight-fitting mouthpiece, and will have nose clips. To minimize the risk, a study team member trained in the procedure performed the test.

AIS (ASIA Impairment Scale) test per ISNCSCI: There is a risk of discomfort at the site of the pin prick during the ASIA Exam. To minimize the risk, a study team member trained in the procedure performed the ASIA exam.

**Functional Reach (FR) test**: Patients may feel anxiety related to loss of balance. To minimize the risk, a study team member trained in the procedure performed the test and will position themselves in the direction of the lean so subject does not fall.

**Level of trunk activation**: Patients may feel anxiety related to loss of balance. To minimize the risk, a study team member trained in the procedure performed the test.

**Grasp/pinch testing**: There are no known risks associated with this measurement.

**Quality of life** was measured using the SCI Quality of Life Questionnaire ( **SCI QL-23**, Siösteen *et al.*, 1990, [Copyright © 1990 Health Care Research Unit, Sahlgrenska University Hospital, Sweden]). The SCI QL-23 is a 23-item health-related quality of life questionnaire (weighted scale, aggregate score: GQOL: overall rating of life situation; FUNC: physical and social limitations; DEPR: distress and depressive feelings; PROB: perceived loss of independence and other issues relating to injury. The scores from each dimension of the SCI QL-23 are a valid measure of subjective health status for individuals with SCI.

The Veterans RAND 36-Item Health Survey ( **VR-36**) was also administered. This routine test is utilized for people with health-related issues. The VR-36 is a health-related quality of life questionnaire (weighted scale, aggregate physical components and metal components scores) [Copyright © Boston University School of Public Health, MA]. 

Each subject followed a course of instruction leading to the necessary mastery of basic sailing technique such that at the end of the course subjects were able to navigate a triangular race course on the simulator’s display. Thereafter, the subjects followed a milestone-based competency course to learn to sail independently on the water at the Accessible Sailing Program at the Downtown Sailing Center (DSC). They were scored based on the ability to perform specific sailing maneuvers (steering predetermined courses, sail trimming, tacking, gybing, mark rounding), and the ability to navigate a simple course around marker buoys (triangular configuration) on the computer screen within a predetermined time ( Table 2). A follow-up questionnaire (see Extended data) was completed to assess the subject’s views about sailing on the water after they completed VSail® training program, as well as the subject’s perceived sailing abilities.

**Table 2.** Scoring system for VSail® Trainer competency evaluation. A score of 50% or more was required to be deemed competent.

Simulator - Sailing competency score sheet				
Sailor ID:		Date:		
Standard Sailing Maneuver		Pass/Fail (✔/ ✘)	Max score	User’s Score
1	Steering crosswind at 90° to wind direction arrows (ability to sail straight course with appropriate sail trim)		(10)	
2	Steering downwind parallel to wind direction arrows (ability to sail straight course with appropriate sail trim)		(10)	
3	Steering upwind (close hauled) on either tack		(10)	
4	Tacking		(10)	
5	Gybing		(10)	
6	Rounding the windward mark		(10)	
7	Rounding the wing mark		(10)	
8	Rounding the leeward (downwind) mark		(10)	
9	Sail around a triangular course in <3 minutes		(20)	
**Total Score:**				

### Statistical analyses

Statistical analysis was limited by the justifiable lack of a control group and the limited number of participants who completed the study (see “Limitations of study” for comment). Each participant was used as their own control and analysis based on a pre-post comparison (t-test) of baseline and test outcome results. The independent variable was time (i.e., Baseline, Postintervention at 6 weeks, postintervention at 12 weeks). The following were dependent variables: (a) ASIA score, (b) composite sailing scores, (c) and aggregate score in each of the four dimensions of the SCI QL-23, and (d) weighted scale, aggregate score in each of the dimensions of the VR-36 is a health-related quality of life questionnaire. All statistical analyses were performed by using Prism GraphPad Statistical Software (Dotmatics, Boston, Massachusetts; https://www.graphpad.com/2023). A p-value of 0.05 or less was considered statistically significant.

## Results

In total, 20 participants were initially recruited for the study; however, seven participants did not complete the entire study. Reasons for withdrawal were, access to transportation and scheduling conflicts with rehabilitation midway through the protocol. Therefore, the final data were included from seven males and six females, six of the participants were tetraplegics and seven participants were paraplegic. Not all of these were able to complete all tasks. The numbers that did complete particular tasks are shown in each Figure and are in Underlying data. The mean age of the participants was 45 years with a range of 23 to 63 years and average time since injury of 14.7 years with a range from 2 to 38 years.

As indicated in Methods while training, the subjects were assessed for fatigue, symptoms, and signs of Autonomic Dysreflexia and symptoms of orthostatic hypotension. None of these were noted during the training sessions. No injuries or other adverse events were reported during the training sessions.

**Figure 3.**
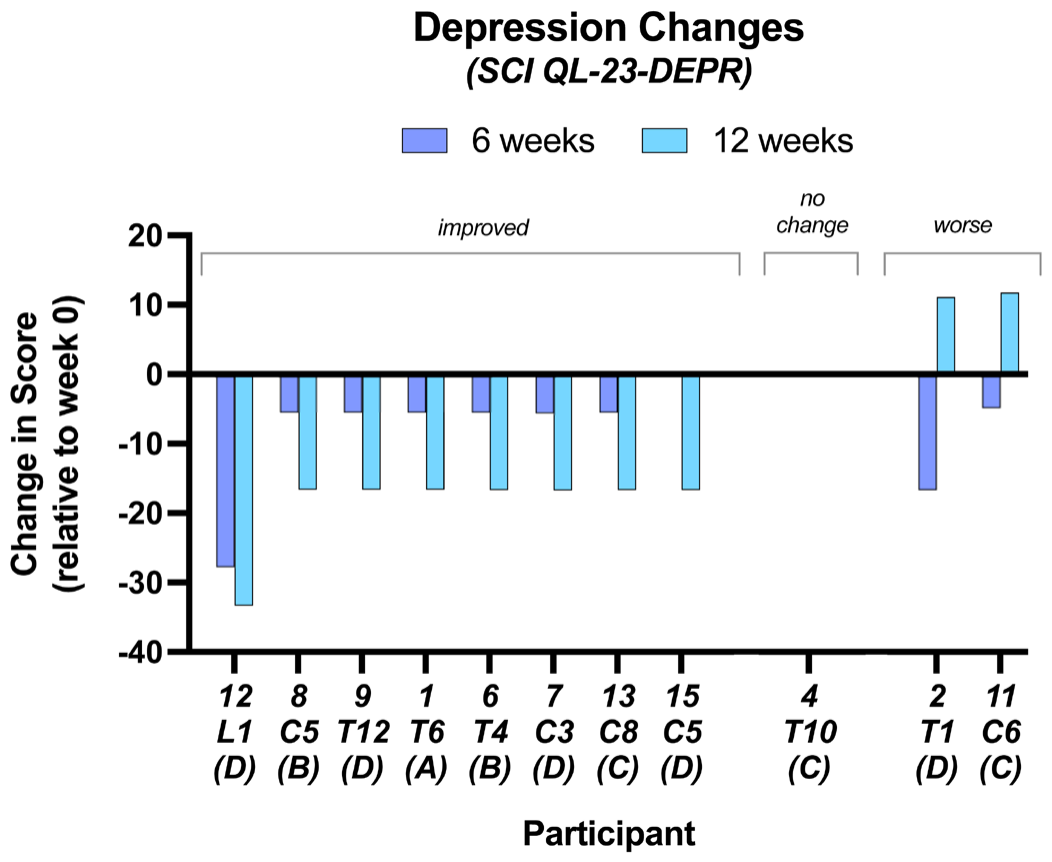
Changes in depression scores. At 6 and 12 weeks compared to baseline. Individual subject scores shown for SCI level and completeness of SCI. Values from SCI QL-23, original data in Underlying data. Note negative numbers on the y axis indicate improvement in depression scores.

For the **depression scores** 7 subjects improved at 6 weeks and were further improved at 12 weeks; an 8 ^th^ subject for which we do not have data at 6 weeks showed an improved score at 12 weeks compared to baseline ( Figure 3). Two improved at 6 weeks but deteriorated at 12 weeks. One subject showed no change. The 6-week scores compared to baseline were significant (p = 0.01) and also at 12 weeks (p = 0.02). There was no significant difference between 6 and 12 weeks (p = 0.045)

**Figure 4.**
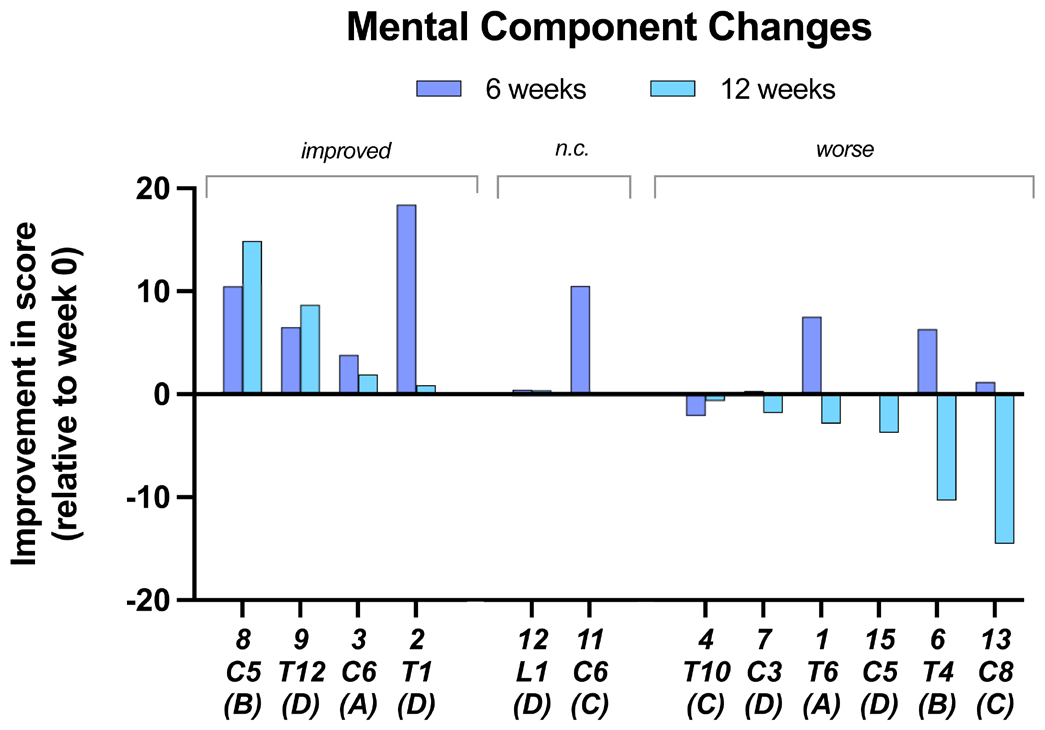
Mental component changes. At 6 and 12 weeks relative to baseline. Individual subject scores are shown for SCI level and SCI completeness score.

In the **mental component change** scores, seven of 12 participants showed an improvement at 6 weeks, but only two improved further at 12 weeks. Two subjects were unchanged at 12 weeks and the rest showed a decrease in score with 6 below baseline ( Figure 4).

**Figure 5.**
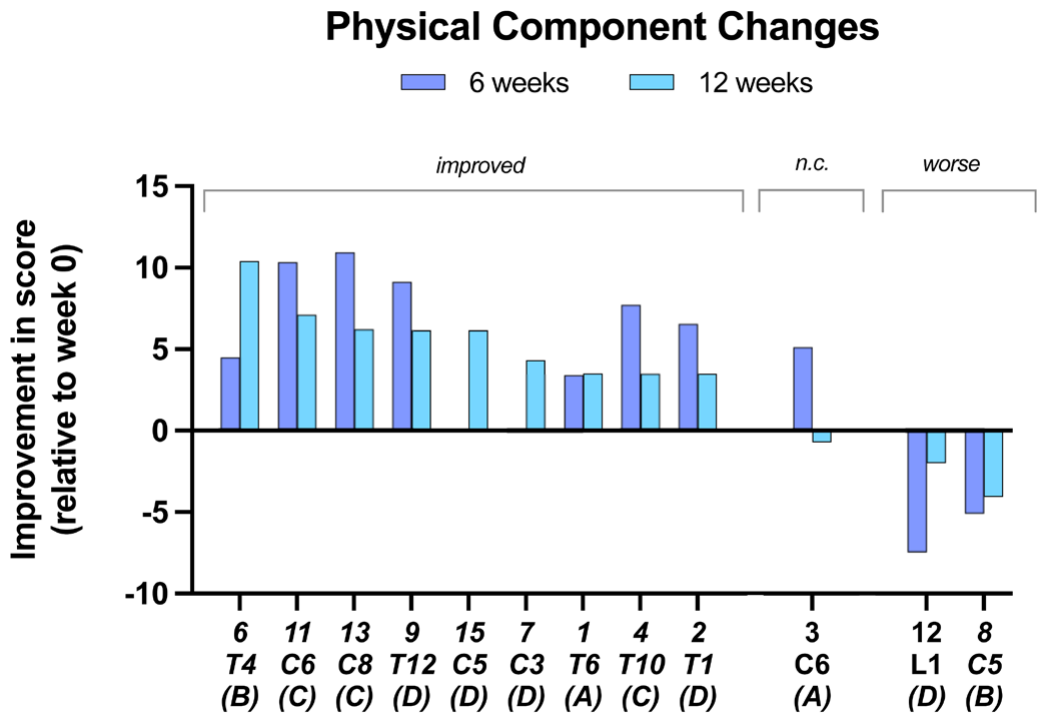
Changes in Physical component scores. At 6 and 12 weeks compared to baseline. Individual subject scores are shown for SCI level and completeness score.

In the **assessment of physical components (VR-36)**, 9 of 12 subjects showed improvement at 12 weeks with the other 3 showing a decline ( Figure 5). Of the improved subjects at 12 weeks only one had improved on their 6-week score.

**Figure 6.**
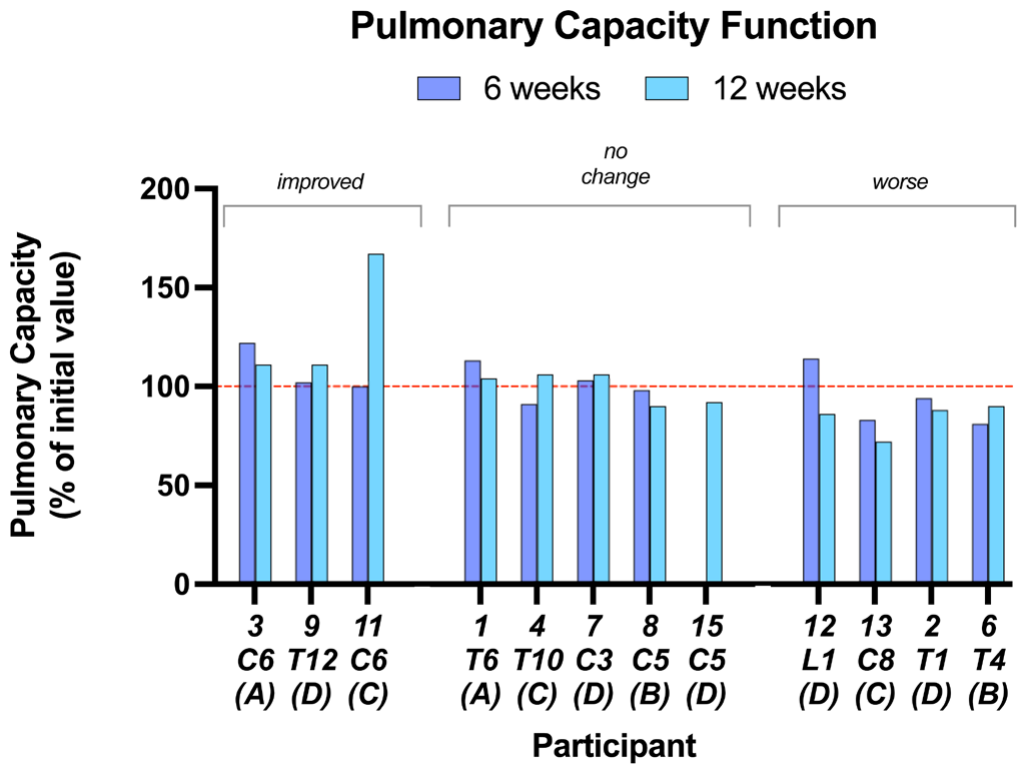
Pulmonary function capacity. This parameter was little changed in most subjects at either 6 or 12 weeks. Individual subject scores are shown for SCI level and completeness score.

Most participants showed little or no change in the various physical measurements made. **Pulmonary function capacity** is shown in Figure 6 as an example. Data on the other measurements are in Underlying data. Five of 12 participants showed no change over baseline in pulmonary capacity function at 6 and 12 weeks. Three improved but only one showed a marked improvement. Four showed relatively small decreases.

**Figure 7.**
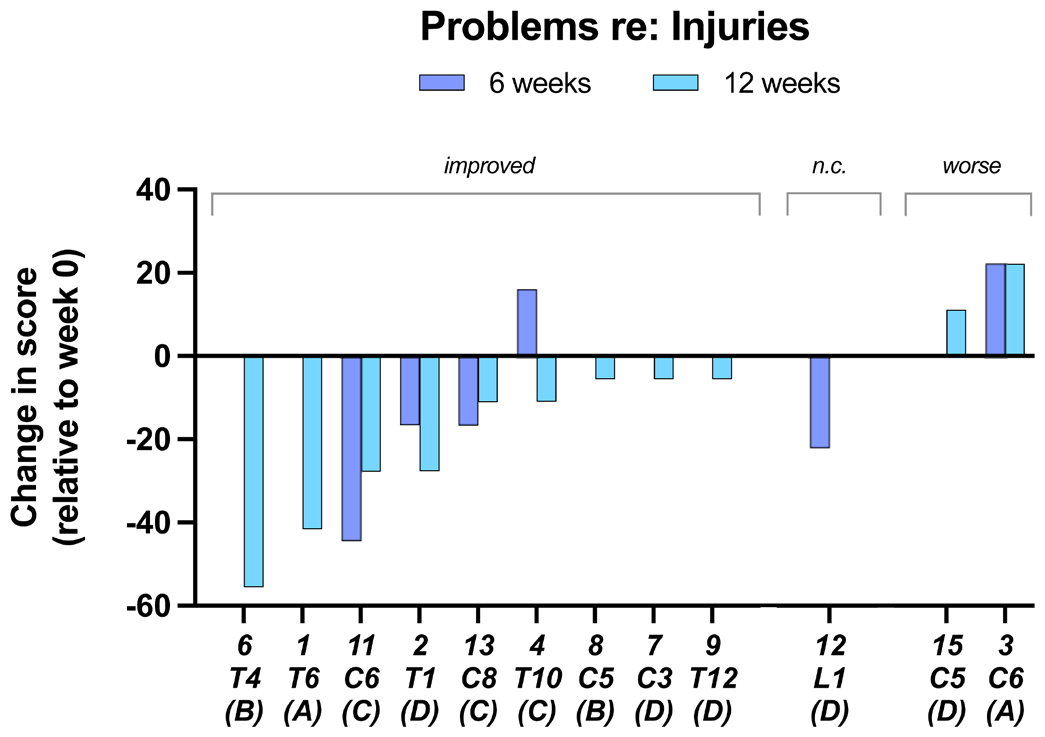
Problems re: injuries. Individual subject scores are shown for SCI level and completeness score. Negative values on the y-axis indicate improvement.

In the results of the questionnaire aimed are determining whether there was a change in the participants perception of any **problems related to their injuries** during the 12-week trial only two reported that there was a deterioration in their injury state ( Figure 7).

**Figure 8.**
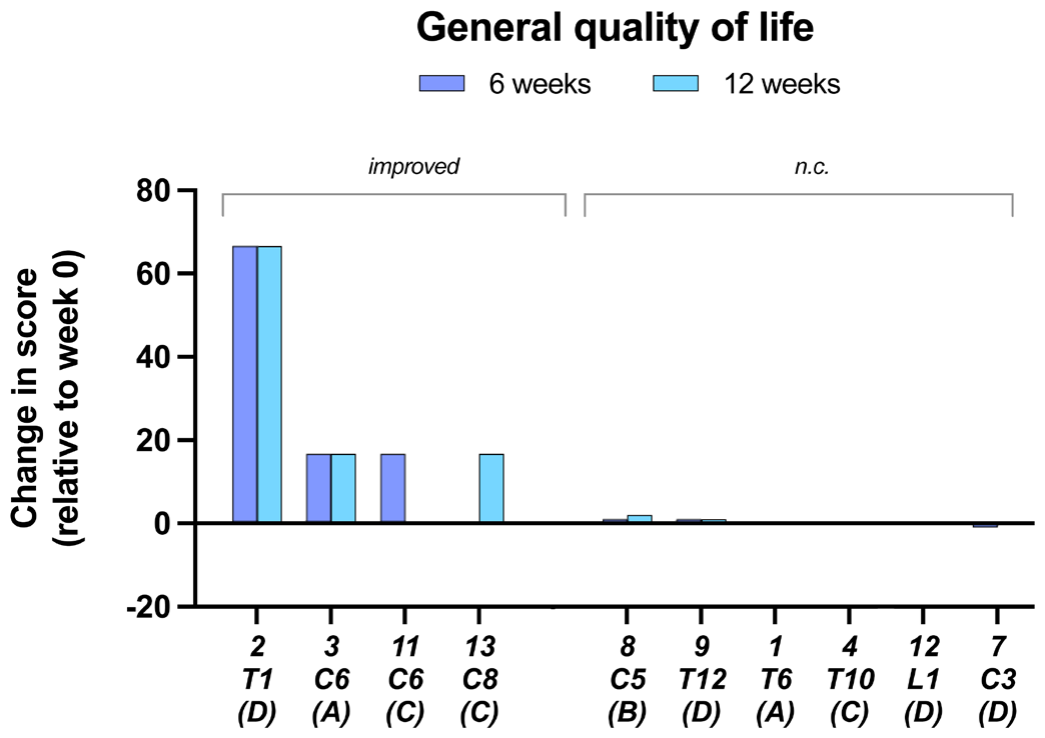
General Quality of life. Data are from SCI QL-23-GQOL changes at 6 and 12 weeks.

In the assessment of the **General quality of life** from SCI QL-23-GQOL four of the ten subjects for which data were obtained showed an improvement ( Figure 8).

Twelve participants completed the 12-week intervention protocol. This included successful completion of a triangular race course in under 3 minutes in 12 knots of wind.

**Figure 9.**
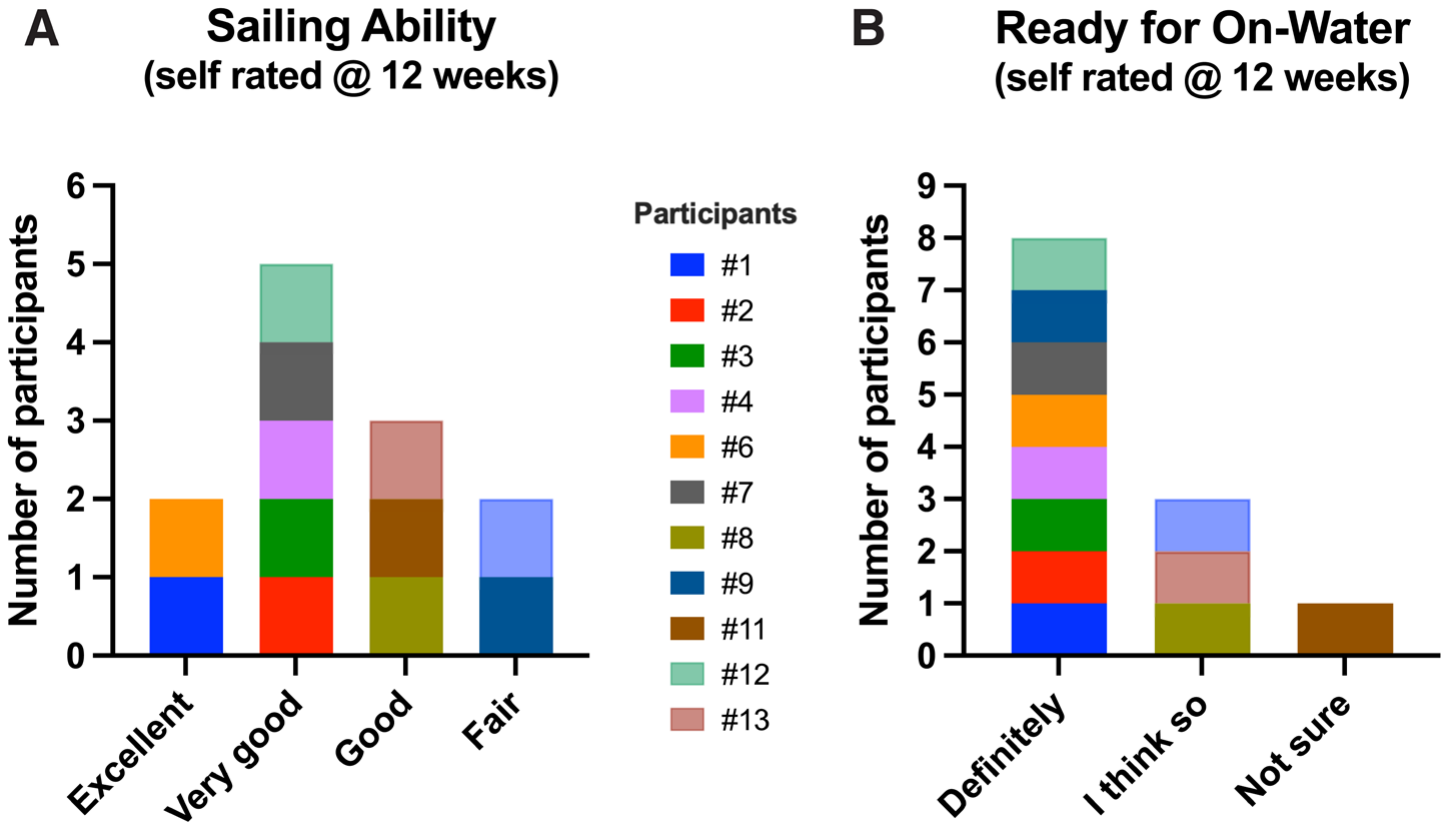
**A**. Self-rating by participants. Simulator sailing ability at 12 weeks. **B**. Self-rating. Readiness to go on water at 12 weeks.

All demonstrated a rapid and substantial improvement in their sailing scores. Passing this competency test meant that the individual performed and understood the basic sailing maneuvers on the VSail® Trainer. When asked about their sailing abilities following the training, 10/12 responded that they were good to excellent. When asked about their readiness to sail on water 11 were confident they were definitely ready or thought they were ready ( Figure 9).

After completion of the twelve-week VSail® training, participants were able to successfully sail adapted sailboats on the water at the Downtown Sailing Center (DSC) in Baltimore. They were able to perform specific sailing maneuvers (i.e., steering predetermined courses, sail trimming, tacking, gybing and mark rounding) after completing the 12-week virtual reality sailing study.

## Discussion

This study provided evidence that people with SCI, using the VSail® technology, could learn to sail achieving a skill level permitting on water sailing. All participants that completed the study protocol were all competent to begin to sail on the water in moderate conditions (wind strength up to 12 knots) and showed measurable improvements in their quality of life, morale, self-esteem, and self-confidence and some aspects of physical health.

There is very little published evidence on the effectiveness of this approach to improving the well-being of people with SCI. We have previously published results of a preliminary trial ( Recio *et al.*, 2013) which suggested that improvements were likely. Since then the results from three trials using a similar protocol have been reported. In an interim report of a trial being conducted in Melbourne, Australia Saunders *et al.* (2016) nine participants after 4–8 simulator sessions were able to sail on their own in 12 or more knots of wind, which was the primary endpoint of the trial. This involved two on-water sessions, one for familiarization and a second that involved sailing round a triangular course. This was evaluated independently by two experienced sailors who had not been involved in the simulator sessions. The participants sailing scores (using the same criteria as in Table 1 above) were similar for the simulator and on-water sessions and ranged from 55-95/100. Two other trials have been published using the VSail® simulator with SCI patients ( Manzanares *et al.*, 2021; Manzanares *et al.*, 2023). Neither of these involved transfer to on-water sailing because the Spinal Injury Centre, National Hospital of Paraplegics of Toledo (Spain) where the patients were tested was too far from the suitable on-water facilities. However, both studies provided evidence of the effectiveness of VSail® technology in proving a range of aspects of the well-being of patients with SCI. Because the protocol did not include an endpoint of sailing on-water in Manzanares *et al.* (2021) it was possible to include a control group. Eleven subjects were randomly assigned to a control group (standard rehabilitation therapy) and a VSail® group (standard therapy plus 30–40 min per day, three times per week for six weeks). Quality of life (QoL), functionality and balance variables were measured for both groups one week before and after the intervention. Significant improvements were obtained in the experimental group in the mobility and balance variables, and in the global result of QoL. In the study of Manzanares *et al.* (2023) six adult patients with SCI underwent VSail® trainer therapy for 40 min sessions, three times per week for six weeks. The training involved learning basic sailing techniques.

Sailing learning, heart rate and effort perception were assessed. Improvements were recorded in these performance variables as well as reduction in the time of the Study taken to sail round a standard race course.

## Limitations of the study

There were several limitations to this study. The most significant was the small sample size of participants recruited. The small number of participants was because of scheduling challenges and staff time availability. Conclusions and information from this study will rather be used as pilot work for a larger study. With this small sample size, results (e.g. mean differences, effect sizes) may not be reflective of actual population parameters. Second, there is a potential risk for the Hawthorne effect ( Landsberger, 1958). Participants in this investigation were aware they were being monitored during the intervention. Therefore, the participants may have been responding to the presence of the research team. Third, there was no formal control group (i.e., those that did not exercise on the simulator) recruited for this study. Recruiting a group of adults with SCI who did not perform any exercise intervention could be detrimental to the health of these individuals, as patients could become deconditioned. Also, an important endpoint was whether or not the participants could sail on water following the VSail® trainer sessions. It would have been unethical to ask patients with SCI to sail on water without any training.

Therefore, it was decided that the study would follow a within subject design and that each participant would act as their own control.

Improvements in the physical parameters were limited, probably because one session a week is insufficient physical activity (compare Manzanares *et al.*, 2021).

People with SCI have variable complications in their lives. This was reflected in the study in that only 13 of the 20 participants completed enough sessions to yield useable data. Although the participants who did complete the 12-week sessions reported no particular injury-related problems it would be difficult to control for any such variation between participants.

## Future studies

Future such studies would be improved by recruiting more participants and randomizing them to a control standard physical therapy group and one of standard therapy plus VSail training several sessions per week, with only the VSail group going on water. The present study took 10 years to complete so a more comprehensive one would require more substantial resources and would probably need to be multi-centre. Participation by more patients could come through utilization during inpatient rehabilitation and location of VSAIL units in outpatient clinics at a distance from SCI centres and using telerehabilitation ( Haig & Stiens, 2017).

## Conclusions

It is generally acknowledged that participation in sports and recreation has a major positive impact on the health and well-being of people with SCI and other disabilities; sailing seems to be particularly favourable in these respects ( Carta *et al.*, 2014; Jenkin & Wilson, 2009; Salvatoni *et al.*, 2003; Sancassiani *et al.* 2017). This study provides evidence that the use of Virtual Sailing’s VSail® technology provides a flexible and adaptable pathway into sailing that is effective in teaching the skills required for on-water sailing for people with a wide range of abilities. The study also provides evidence that this approach to rehabilitation can yield improvements in a number of physical and mental attributes, including QoL. This study involved only people with SCI, but is also being developed in other centres for people with other disabilities and social disadvantage, e.g. Carta *et al*. (2014); Mooney *et al*. (2009); Sancassiani *et al.* (2017).

## Data Availability

Monash University: Reico *et al.* Underlying data.xlsx, https://doi.org/10.26180/24821421 ( Habgood & Saunders, 2023a). Monash University: Reico *et al.* Extended data.pdf, https://doi.org/10.26180/24821589 ( Habgood & Saunders, 2023b). Data are available under the terms of the Creative Commons Attribution 4.0 International license (CC-BY 4.0). ClinicalTrials.gov: NCT01491789 Protocol ID: NA_00044093 Approval date: 5/5/2011 Termination Date: 9/12/2023
