# Supplementary material for: Realtime physical simulator for virtual reality sailing by patients with spinal cord injury: an innovative voyage
Source: Health Open Res. 2024 Sep 11;6:6. Originally published 2024 Feb 16. [Version 2] doi: 10.12688/healthopenres.13582.2 (PMC11450545; doi:10.12688/healthopenres.13582.2)
Supplement: Peer review history

# Reviewer report: Reviewer response for version 2

**DOI:** 10.21956/healthopenres.14857.r28815

**Published:** 2024-10-14

**Authors:** Aarón Manzanares Serrano

**Affiliations:** 1 Universidad Catolica San Antonio de Murcia, Murcia, Region of Murcia, Spain

The authors have made the requested modifications and suggestions.

Is the work clearly and accurately presented and does it cite the current literature?

Yes

If applicable, is the statistical analysis and its interpretation appropriate?

Yes

Are all the source data underlying the results available to ensure full reproducibility?

Yes

Is the study design appropriate and is the work technically sound?

Yes

Are the conclusions drawn adequately supported by the results?

Yes

Are sufficient details of methods and analysis provided to allow replication by others?

Partly

Reviewer Expertise:

Sailing; Virtual Reality; Spinal cord injury

I confirm that I have read this submission and believe that I have an appropriate level of expertise to confirm that it is of an acceptable scientific standard.

# Reviewer report: Reviewer response for version 2

**DOI:** 10.21956/healthopenres.14857.r28814

**Published:** 2024-10-11

**Authors:** Nidhi Sharma, Parveen Kumar

**Affiliations:** 1 Maharishi Markandeshwar Institute, Mullana, India; 2 Pal Healthcare, Pal Physiotherapy and Rehabilitation Centre, Ambala City, Haryana, India

I have carefully reviewed the revised manuscript submitted by the author. While I would like to commend the author for their diligent efforts in improving the manuscript, it is important to note that not all the points raised in the initial review have been fully acknowledged or addressed.

 However, despite these omissions, the revisions made reflect substantial improvements to the manuscript. The core scientific content has been enhanced, and the clarity of the presentation is now of a high standard. The overall quality of the work is satisfactory, and in my opinion, it meets the journal's requirements for approval.

 Considering the significant progress made in the revised version, I recommend that the manuscript be approved for indexing.

Is the work clearly and accurately presented and does it cite the current literature?

Partly

If applicable, is the statistical analysis and its interpretation appropriate?

No

Are all the source data underlying the results available to ensure full reproducibility?

No

Is the study design appropriate and is the work technically sound?

No

Are the conclusions drawn adequately supported by the results?

Partly

Are sufficient details of methods and analysis provided to allow replication by others?

No

Reviewer Expertise:

methodology, rehabilitation, outcome measures, statistics, results

We confirm that we have read this submission and believe that we have an appropriate level of expertise to confirm that it is of an acceptable scientific standard.

# Response

**Published:** 2024-10-11

**Authors:** Norman Saunders

**Affiliations:** Neuroscience, Monash University, Melbourne, Victoria, Australia

I thank Dr. Sharma for his comments on the revised manuscript and his approval of this version.

 Norman Saunders

# Reviewer report: Reviewer response for version 2

**DOI:** 10.21956/healthopenres.14857.r28829

**Published:** 2024-10-01

**Authors:** Junhua Xiao, Radietya Alvarabie

**Affiliations:** 1 Swinburne University of Technology, Hawthorn,, VIC, Australia; 2 Swinburne University of Technology, Hawthorn, Victoria, Australia

The study focuses on a unique and innovative application of virtual reality (VR) and real-time sailing simulators for spinal cord injury (SCI) patients. It addresses the integration of physical rehabilitation with immersive VR technology, which is both novel and beneficial in rehabilitation science. The study demonstrates tangible improvements in participants’ physical and psychological well-being, including a decrease in depression and an increase in self-esteem, morale, and quality of life, suggesting the potential of virtual reality-based sports rehabilitation for people with disabilities, fostering greater community reintegration through recreational activities

 The methods section is well-detailed, outlining the inclusion criteria, physical and psychological assessments, and the structure of the training sessions. This structure ensures replicability for future studies. The paper provides thorough outcome data, using various metrics such as ASIA scores and quality of life assessments, making the study valuable for practitioners aiming to understand the effects of simulator training.

 The study involved various experts from different fields, which adds depth to the analysis and ensures a more comprehensive understanding of the subject.

 A post-intervention follow-up study that Focuses on Long-Term Outcomes would provide additional insights into the lasting impact of the VSail intervention. Therefore, this point should be discussed in Future Studies to improve the manuscript.

Is the work clearly and accurately presented and does it cite the current literature?

Yes

If applicable, is the statistical analysis and its interpretation appropriate?

Yes

Are all the source data underlying the results available to ensure full reproducibility?

Yes

Is the study design appropriate and is the work technically sound?

Yes

Are the conclusions drawn adequately supported by the results?

Yes

Are sufficient details of methods and analysis provided to allow replication by others?

Yes

Reviewer Expertise:

neuroscience, digital health

We confirm that we have read this submission and believe that we have an appropriate level of expertise to confirm that it is of an acceptable scientific standard.

# Reviewer report: Reviewer response for version 1

**DOI:** 10.21956/healthopenres.14669.r27896

**Published:** 2024-04-25

**Authors:** Devi Nampiaparampil

**Affiliations:** 1 New York University School of Medicine, New York, USA

This study addresses an important topic: it assesses changes in health and quality of life in people with complete and incomplete paraplegia and quadriplegia who used a VSail stimulator to learn how to sail in a safe controlled environment. This relates to virtual reality and learning through the use of technology. It also assess how more patients can exercise and how they can better integrate into their communities. 

 Prior studies have shown that virtual reality systems alone can generate psychological benefits for patients in rehabilitation therapy. Several studies have shown that small boat open water sailing, in particular, improves self-esteem and enhances general health in those with physical or psychological disabilities. 

 There were twenty participants at the start of the study. Thirteen completed it. These individuals undertook a 12-week program to learn how to sail. At 6 weeks post-training completion, the majority of subjects displayed a decrease in depression as well as in their physical and social limitations, and an improvement in their physical assessments. The data was collected over the course of 10 years.

 The researchers used appropriate measures for physical and psychological well-being. The statistical analysis seems reasonable. 

 The authors have written the paper in clear, concise language. The photo and figures are helpful. 

**In terms of areas for improvement–**

1. Perhaps the authors could include a couple screenshots of the training (what the subject sees) and a general idea of how long it took for the subjects to complete each training session and how many sessions (or hours of training) might be needed to achieve competency overall.
2. The authors could provide more information about how practical sailing is for patients with spinal cord injuries. Do patients have to go to specialized hospital-affiliated sites and cities once they achieve competency or can patients get on any boat anywhere?
3. In either the background or the conclusion, perhaps the authors could provide a couple references of how virtual reality has been used to improve performance in sports and recreation in people without disabilities.

Overall, this is an excellent paper and I strongly recommend it for an approval.

Is the work clearly and accurately presented and does it cite the current literature?

Yes

If applicable, is the statistical analysis and its interpretation appropriate?

Yes

Are all the source data underlying the results available to ensure full reproducibility?

Yes

Is the study design appropriate and is the work technically sound?

Yes

Are the conclusions drawn adequately supported by the results?

Yes

Are sufficient details of methods and analysis provided to allow replication by others?

Yes

Reviewer Expertise:

Spinal disorders and spinal cord injuries, chronic pain, procedural techniques, disability

I confirm that I have read this submission and believe that I have an appropriate level of expertise to confirm that it is of an acceptable scientific standard.

# Response

**Published:** 2024-08-07

**Authors:** Norman Saunders

**Affiliations:** Neuroscience, Monash University, Melbourne, Victoria, Australia

We thank Dr Nampiaparampil for approving our paper and for his suggestions on how it could be improved. Screenshots of the training (what the subject sees) are in the VSail Lesson Plan which has been added to the material deposited online (Extended data). Each session lasted about one hour. This has been added to the Methods section.  

 Facilities for people with disabilities to sail are available in many countries. We have added some websites on this.  

 As pointed out by Dr Nampiaparampil virtual reality has been used to improve performance in sports and recreation in people without disabilities. However, this is a substantial topic that is outside of the scope of the report on our study. We have added a recent review by Xiao (2024) which dealt with Virtual Sailing as an Exercise therapy but also mentions a few examples of the use of virtual reality in people without disabilities.

# Response

**Published:** 2024-08-13

**Authors:** Norman Saunders

**Affiliations:** Neuroscience, Monash University, Melbourne, Victoria, Australia

We thank Dr. Nampiaparampil for approving our paper and for his suggestions on how it could be improved.

 Screenshots of the training (what the subject sees) are in the VSail Lesson Plan which has been added to the material deposited online (Extended data). Each session lasted about one hour. This has been added to the Methods section. 

 Facilities for people with disabilities to sail are available in many countries. We have added some websites on this. 

 As pointed out by Dr. Nampiaparampil virtual reality has been used to improve performance in sports and recreation in people without disabilities. However, this is a substantial topic that is outside of the scope of the report on our study.

 We have added a recent review by Xiao (2024) which dealt with Virtual Sailing as an Exercise therapy but also mentions a few examples of the use of virtual reality in people without disabilities.

# Reviewer report: Reviewer response for version 1

**DOI:** 10.21956/healthopenres.14669.r27967

**Published:** 2024-04-03

**Authors:** Nidhi Sharma

**Affiliations:** 1 Maharishi Markandeshwar Institute, Mullana, India

Thank you for inviting me as a reviewer to review this manuscript which provides a new insight for the research and the clinicians as well.

 Here are my additional points which sought clarification - 

 1. Abstract - Age group and Male to female ratio can be mentioned in the Methods Section. Outcome measures must be mentioned, and duration of the protocol must be clearly mentioned. In the Result section, improvement/s must be depicted in terms of Level of Significance. Keywords are not according to MeSH terms.

 2. Introduction was well-written but somewhat lengthy.

 3. Methods- Study design must be clearly mentioned. Authors have included age group 18-80 years. This is a very vast age group; how can u match the baseline characteristics of 18 yrs. old with that of 80 years old. There must be various neuro-musculoskeletal degeneration changes occur in the geriatric population. 

 4. The inclusion criteria is very vague. Exclusion criteria has not been mentioned. What about the associated conditions which are considered to be medically stable like degenerative conditions or muscle wasting due to ageing?

 5. The groups must be clearly mentioned in the text. The demographic characteristics of the patients must be taken into consideration.

 6. Regarding the study intervention, and Figure 1, how the sailing has been performed by patients with quadriplegia. Rigor in Intervention technique is lacking which make it hardly reproducible for the future researchers.

 7. None of the outcome measure was cited and at least, reliability and validity of respective outcome measure for SCI must be mentioned.

 8. Statistical analysis - Repeated Measure ANOVA must be the choice of test, and not the t-test. Two-way ANOVA should also be used to determine the time*group interaction effect. It should also mention the level of significance.

 9. Results- Average time of injury was found to be 14.7 years with a range of 2 to 38 years. It is again a very vast difference between the time elapsed since injury. Definitely patient who was injured 38 years ago, must show good recovery in comparison to the patient of 2 years injury. None of the table show the improvement in terms of level of significance.

 10. Discussion - When the authors have published the preliminary trial, then how could this trial be a 'Pilot trial'?

 The study has a lot of potential to improve in its methodology, statistics and results. The authors must acknowledge the concerns before proceeding further.

 Thanks

Is the work clearly and accurately presented and does it cite the current literature?

Partly

If applicable, is the statistical analysis and its interpretation appropriate?

No

Are all the source data underlying the results available to ensure full reproducibility?

No

Is the study design appropriate and is the work technically sound?

No

Are the conclusions drawn adequately supported by the results?

Partly

Are sufficient details of methods and analysis provided to allow replication by others?

No

Reviewer Expertise:

methodology, rehabilitation, outcome measures, statistics, results

I confirm that I have read this submission and believe that I have an appropriate level of expertise to state that I do not consider it to be of an acceptable scientific standard, for reasons outlined above.

# Response

**Published:** 2024-08-07

**Authors:** Norman Saunders

**Affiliations:** Neuroscience, Monash University, Melbourne, Victoria, Australia

We thank Dr. Sharma for his comments and suggestions for improvements to our paper, some of which we are able to deal with. However, we have had to take account of the complexity of sailing as a task both on the simulator and on the water and the variability in the population of people with spinal cord injuries we were able to recruit for the study. The characteristics of the study population are now included in a Table (1) in the Methods section as suggested by Dr Manzanares (points 1 and 5).  

 The main outcome measure is clearly stated in the Abstract Conclusion in the form of the primary objective. The study design is outlined in Fig. 2 (point 3). The exclusion criteria were those that did not meet the inclusion criteria (point 4). It is unclear what is meant by “Rigor ( *sic*) in Intervention technique is lacking” (point 6).  

 The outcome measures are clearly the results of the various tests; it seems unnecessary to spell this out separately. There have been very few studies of this kind but the data obtained will provide evidence of validity of the outcome measures for future studies (point 7).  

 The statistical analysis has been revised but does not make any difference to the conclusions drawn from the results.  (Point 8).  

 The study was designated as a “Pilot” (point 10) as we are not so bold as to claim it is definitive and hope that it will lead to future studies.

# Response

**Published:** 2024-08-13

**Authors:** Norman Saunders

**Affiliations:** Neuroscience, Monash University, Melbourne, Victoria, Australia

We thank Dr. Sharma for her comments and suggestions for improvements to our paper, some of which we are able to deal with. However, we have had to take account of the complexity of sailing as a task both on the simulator and on the water and the variability in the population of people with spinal cord injuries we were able to recruit for the study.

 The characteristics of the study population are now included in a Table (1) in the Methods section as suggested by Reviewer 1 - Dr. Manzanares (points 1 and 5).

 The main outcome measure is clearly stated in the Abstract Conclusion in the form of the primary objective. The study design is outlined in Fig. 2 (point 3). The exclusion criteria were those that did not meet the inclusion criteria (point 4). It is unclear what is meant by “Rigor ( *sic*) in Intervention technique is lacking” (point 6).  

 The outcome measures are clearly the results of the various tests; it seems unnecessary to spell this out separately. There have been very few studies of this kind but the data obtained will provide evidence of validity of the outcome measures for future studies (point 7).   

 The statistical analysis has been revised but does not make any difference to the conclusions drawn from the results.  (point 8).  

 The study was designated as a “Pilot” (point 10) as we are not so bold as to claim it is definitive and hope that it will lead to future studies.

# Reviewer report: Reviewer response for version 1

**DOI:** 10.21956/healthopenres.14669.r27898

**Published:** 2024-03-27

**Authors:** Aarón Manzanares Serrano

**Affiliations:** 1 Universidad Catolica San Antonio de Murcia, Murcia, Region of Murcia, Spain

I would like to congratulate the authors for their work. There is great value in doing research that takes people with spinal cord injury into the water. It also presents a very large sample in relation to the study population, which makes it a very valuable research.

 The introduction is good and concise, focusing on the objective of the study. While doing research on real and simulated sailing and the learning process, I recommend including the following research.[1]

 In relation to the description of the sample, I would appreciate more specific information on the age of the subjects, gender, time since the injury or on pathologies associated with the injury and not associated with the injury. I see that some of this information is in the results section and based on the subjects who finally completed the research, but I think it would be appropriate to move it to the method section. I believe that given the results and the statistics used, this would give greater value to the research.

 With regard to the methodology used for teaching navigation in a simulator and then in a real navigation situation, could you please specify the contents and exercises carried out in each session? I am aware that each person may have a different learning pace and therefore the same activity is not strictly followed for all subjects. Despite this, I would appreciate it if the article could include the script that was followed in each session in terms of content and exercises used.

 In the limitations section they make the following statement "Improvements in the physical parameters were limited, probably because one session a week is insufficient physical activity (compare Manzanares et al., 2021)". From my point of view, it would be interesting to address this in the discussion section, along with the rest of the variables evaluated in which not all subjects improved after the intervention. In this sense, my attention is not drawn to the subjects who did not improve, but rather to those who obtained worse results after the intervention. This is why I believe that these results should be justified in the discussion section.

 I reiterate my congratulations to the authors for their work.

Is the work clearly and accurately presented and does it cite the current literature?

Yes

If applicable, is the statistical analysis and its interpretation appropriate?

Yes

Are all the source data underlying the results available to ensure full reproducibility?

Yes

Is the study design appropriate and is the work technically sound?

Yes

Are the conclusions drawn adequately supported by the results?

Yes

Are sufficient details of methods and analysis provided to allow replication by others?

Partly

Reviewer Expertise:

Sailing; Virtual Reality; Spinal cord injury

I confirm that I have read this submission and believe that I have an appropriate level of expertise to confirm that it is of an acceptable scientific standard, however I have significant reservations, as outlined above.

## References

[1] : The effect of contextual interference on the learning of adapted sailing for people with spinal cord injury. *Adaptive Behavior* .2022;30(1) : 10.1177/1059712320946110 37-50 10.1177/1059712320946110

# Response

**Published:** 2024-04-24

**Authors:** Albert Recio

**Affiliations:** Kennedy Krieger Institute, USA

We thank the reviewer and highly appreciate the recommendations to improve this manuscript and future VSAIL research.

**The introduction is good and concise, focusing on the objective of the study. While doing research on real and simulated sailing and the learning process, I recommend including the following research.**

**1) In relation to the description of the sample, I would appreciate more specific information on the age of the subjects, gender, time since the injury or on pathologies associated with the injury and not associated with the injury. I see that some of this information is in the results section and based on the subjects who finally completed the research, but I think it would be appropriate to move it to the method section. I believe that given the results and the statistics used, this would give greater value to the research.**

**Response: **The age range and gender are all indicated and stated in the Methodology section. The majority of the patients are males with an age range of 23 to 63 years. Just like in the National average, majority of Spinal cord injury patients were males. The years from injury was included in this study to demonstrate that these are all chronic injuries and the improvements made as a result of the study are not occurring by spontaneous recovery. The average length of injury is included in the Results section which indicates that it is more than 6 months and that they are chronic.

**2) With regard to the methodology used for teaching navigation in a simulator and then in a real navigation situation, could you please specify the contents and exercises carried out in each session? I am aware that each person may have a different learning pace and therefore the same activity is not strictly followed for all subjects. Despite this, I would appreciate it if the article could include the script that was followed in each session in terms of content and exercises used.**

**Response:** The lesson plans were made basic and were the same across all subjects. We could add the lesson plan as an appendix. We would like to remind the readers that this is a Pilot Study and as such the lesson plans were not included. We are planning on publishing that as a separate Manuscript for the next VSAIL research.

**3) In the limitations section they make the following statement "Improvements in the physical parameters were limited, probably because one session a week is insufficient physical activity (compare Manzanares et al., 2021)". From my point of view, it would be interesting to address this in the discussion section, along with the rest of the variables evaluated in which not all subjects improved after the intervention. In this sense, my attention is not drawn to the subjects who did not improve, but rather to those who obtained worse results after the intervention. This is why I believe that these results should be justified in the discussion section.**

**Response: **The aim of the study is to show the value of the VSAIL as a tool for teaching how to sail. In the interest of recruiting participants, we are unable to increase the number of hours and frequency to affect increase in function. It is established that improvements in SCI takes time and is a slow process. Rehabilitation protocols are recommending two to three times a week to improve function, which is not doable with a VSAIL.

**I reiterate my congratulations to the authors for their work.**

# Response

**Published:** 2024-08-08

**Authors:** Norman Saunders

**Affiliations:** Neuroscience, Monash University, Melbourne, Victoria, Australia

We would like to thank Dr Manzanares Serrano for his supportive and helpful review of our paper. In response to his suggestions we have incorporated a Table of data on the participants, which is now in the Methods section. It replaces the current Table 1 as the data in that Table are now in the new Table. We have added a file of the Lesson Plan to the material deposited online. Comments about the variation in the level of change in each test have been added to the Discussion as requested.

# Response

**Published:** 2024-08-13

**Authors:** Norman Saunders

**Affiliations:** Neuroscience, Monash University, Melbourne, Victoria, Australia

We would like to thank Dr Manzanares Serrano for his supportive and helpful review of our paper. In response to his suggestions we have incorporated a Table of data on the participants, which is now in the Methods section. It replaces the current Table 1 as the data in that Table are now in the new Table. We have added a file of the Lesson Plan to the material deposited online. Comments about the variation in the level of change in each test have been added to the Discussion as requested.
